# The Role of Cardiac Magnetic Resonance in the Evaluation of Patients Presenting with Suspected or Confirmed Acute Coronary Syndrome

**DOI:** 10.4061/2011/605785

**Published:** 2011-10-19

**Authors:** Loren P. Budge, Michael Salerno

**Affiliations:** ^1^Cardiology Division, Department of Medicine, University of Virginia Health System, 1215 Lee Street, P.O. Box 800158, Charlottesville, VA 22908, USA; ^2^Department of Radiology, University of Virginia Health System, 1215 Lee Street, P.O. Box 800158, Charlottesville, VA 22908, USA; ^3^Department of Biomedical Engineering, University of Virginia Health System, 1215 Lee Street, P.O. Box 800158, Charlottesville, VA 22908, USA

## Abstract

Cardiac magnetic resonance imaging (CMR) has an important emerging role in the evaluation and management of patients who present with symptoms concerning for acute coronary syndrome (ACS). This paper discusses the role of CMR in the emergency department setting, where CMR can aid in the early and accurate diagnosis of non-ST elevation ACS in low and intermediate risk patients. For those with confirmed myocardial infarction (MI), CMR provides comprehensive prognostic information and can readily diagnose structural complications related to MI. Furthermore, the pattern of late gadolinium enhancement (LGE) seen on CMR can help determine the etiology of cardiac injury in the subset of patients presenting with ACS who do not have obstructive coronary artery disease by angiography.

## 1. Introduction

Currently, more than a third of the 5.5 million people who present to the emergency department (ED) annually in the United States with a chief complaint of chest pain are admitted to the hospital, while only about a third of those admitted are eventually given the diagnosis of acute coronary syndrome (ACS) [1, 2]. Thus, the emergency department physician is confronted on a daily basis with patients who have symptoms worrisome for possible ACS. Available tools in the ED, which include a detailed medical history, physical exam, ECG, and serum cardiac biomarkers, have limitations for accurately and rapidly diagnosing ACS. Cardiac troponin I/T are quite sensitive for detecting myocardial injury, but often do not become elevated until hours after an infarction has occurred and may not be elevated in ACS without myocardial infarction. Triaging of these patients may be straightforward for high-risk patients, especially those with positive serum biomarkers (troponin I or T or myocardial fraction of creatine kinase) or with evidence of myocardial infarction (MI) on an electrocardiogram (ECG). However, for low- and intermediate-risk patients without immediate evidence of ischemia or MI on presentation, the decision of whether to admit can often be challenging. Furthermore, there is evidence that a small, but clinically significant number of patients are discharged from the ED with a missed diagnosis of ACS, including MI [3]. Missing the diagnosis of ACS and inappropriately discharging patients can result in adverse patient outcomes and is the leading cause of malpractice lawsuits and verdicts for physicians in the emergency room [4]. Because of the high prevalence and mortality associated with ACS, long observation times or admission to the hospital is often required. Even after patients have “ruled out” for MI, further functional or anatomic testing is frequently performed for risk stratification. An ideal test for ACS in the ED would be able to quickly, accurately, and noninvasively triage patients with ACS symptoms shortly after arrival to the ED. Cardiac magnetic resonance imaging (CMR) is proving to be a powerful tool for the early identification or exclusion of ACS as the cause of a patient's chest pain syndrome. Patients with evidence of MI on presentation to the ED often undergo an early invasive strategy consisting of cardiac catheterization with potential percutaneous coronary intervention (PCI). In this setting, CMR, performed after the intervention, has been shown to provide valuable prognostic information. Patients at low risk for ACS following CMR examination can potentially be discharged, thus reducing cost from unnecessary hospital admission. Additionally, for the challenging subset of patients with evidence of MI on presentation, but who have normal coronary arteries at catheterization, CMR is uniquely able to identify the correct etiology in the majority of cases. This paper will discuss the role of CMR in the early evaluation of patients presenting to the ED with symptoms concerning for ACS, the role of CMR after MI, and the role of CMR for assessing patients presenting with ACS who are found to have nonobstructive CAD by cardiac catheterization.

## 2. CMR Techniques for Evaluating ACS

CMR is capable of performing a rapid and comprehensive evaluation of cardiac anatomy, function, and myocardial perfusion at rest and/or during stress. CMR can accurately identify the presence of infarction, myocardial edema, microvascular obstruction, and intramyocardial hemorrhage. The sequence of a typical CMR exam for ACS usually begins with anatomic images of the chest, which can often provide clues about other pathologies that may be causing the patient's chest pain. Cine steady-state free precession imaging provides an assessment of myocardial and valvular function and enables accurate assessment of left ventricular ejection fraction (LVEF) and ventricular volumes. CMR can assess myocardial edema and inflammation using *T*_2_-weighted imaging due to the increased water content of the myocardium. Edematous tissues with higher water content have longer *T*_2_ relaxation times, leading to a brighter signal on *T*_2_-weighted images. First-pass myocardial perfusion is assessed using saturation-recovery gradient echo techniques during intravenous injection of gadolinium contrast agents. This can be performed during pharmacologic stress using adenosine or dobutamine infusion providing an assessment of myocardial ischemia. Myocardial scar is assessed by late gadolinium-enhanced (LGE) imaging, the pattern of which can help differentiate infarction from nonischemic processes. Regions with myocardial scar have a higher concentration of contrast agent and, thus, appear bright in these images. Conversely, areas of microvascular obstruction, thrombus, and myocardial hemorrhage appear dark on LGE imaging. A complete CMR protocol can be performed in less than 45 minutes providing a rapid comprehensive assessment of the heart.

## 3. Deciding Whether to Admit

CMR has been shown to improve both the diagnostic accuracy and time to diagnosis of ACS in the emergency department setting. This was demonstrated in a study of 161 patients presenting to the ED within 12 hours of onset of symptoms concerning for ACS, but without ST elevation on ECG [5]. The CMR exam in this study included cine MRI for LV function, resting first-pass perfusion, and LGE. Total CMR time was 38 minutes, and patients were gone from the ED for less than 1 hour. The sensitivity and specificity for diagnosis of ACS by CMR was 84% and 85%, respectively, and added independent diagnostic value to clinical parameters [5]. The main limitation of CMR in this study was its inability to differentiate acute from chronic MI, a problem which was addressed in a subsequent study by Cury et al., who added *T*_2_-weighted imaging and LV wall thickness quantification to distinguish acute events [6]. *T*_2_-weighted imaging enables visualization of edema associated with acute, but not chronic, myocardial infarction [7]. In this study, 62 patients with chest pain, normal ECG, and negative initial cardiac biomarkers, who were being admitted to the hospital to rule out MI, received CMR with an average scan time of 32 minutes. CMR with *T*_2_-weighted imaging accurately identified all acute MI cases, often before cardiac biomarkers became elevated. Compared with conventional CMR, the addition of *T*_2_-weighted edema imaging and wall thickness quantification increased specificity from 84% to 96%, positive predictive value from 55% to 85%, and overall accuracy from 84% to 93%; respectively, while sensitivity remained unchanged at 85% [6]. 

## 4. CMR without Initial Evidence of MI

**Figure 1:**
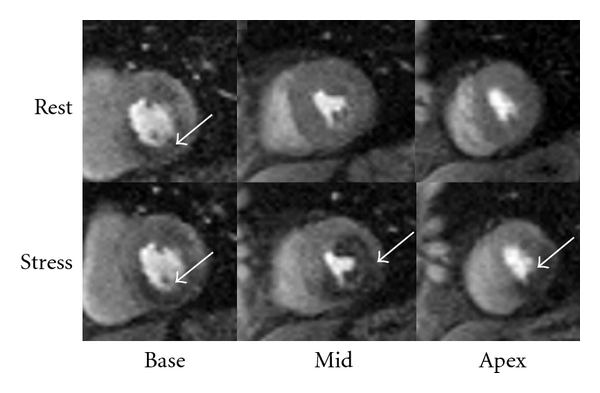
CMR with pharmacologic stress. An example of an adenosine stress CMR in a diabetic patient presenting with new onset chest pain. Resting first-pass perfusion images (top panels) are shown above the corresponding stress perfusion images (bottom panels) in the basal, midventricular, and apical short-axis views. There are inferior and anterior resting perfusion defects in the basal short-axis slice, but with stress this patient develops a severe inferior perfusion defect extending to the lateral wall and septum in the midventricle and apex. On cardiac catheterization (not shown), this patient had multivessel CAD.

A large proportion of low- and intermediate-risk patients presenting to the ED with symptoms concerning for ACS without evidence of MI undergo additional functional or anatomic imaging on either an inpatient or outpatient basis. CMR has been shown to compare favorably to other non-invasive cardiac imaging modalities for diagnosing ischemia. In a study by Nagel et al., 208 patients with suspected CAD underwent both dobutamine stress echo and dobutamine stress CMR prior to cardiac catheterization [8]. With CMR, sensitivity and specificity for detecting a 50% coronary stenosis was increased from 74.3% to 86.2% and from 69.8% to 85.7%, respectively, compared with echocardiography. In a study of 163 patients with poor acoustic windows preventing adequate imaging by dobutamine stress echo, Hundley et al. successfully performed dobutamine stress MRI in 153 of the patients [9]. The sensitivity and specificity for detecting a 50% coronary stenosis were 83% and 83%, respectively, in the 41 patients undergoing coronary angiography within 6 months of their stress test. In the 103 patients with negative stress tests, the event free survival at a median followup of 228 days was 97% [9]. A meta-analysis of dobutamine stress CMR including 14 studies (754 patients) with a high prevalence of coronary disease (prevalence: 70.5%) demonstrated a sensitivity of 83% and specificity of 86% [10]. Adenosine stress cardiac MRI has also been shown to be both sensitive and specific for detection of CAD. A meta-analysis including 1658 patients with intermediate likelihood of disease undergoing adenosine stress perfusion MRI demonstrated a sensitivity of 90% and specificity of 81%. The negative likelihood ratio of adenosine stress in this study was 0.14 [11]. A recent multicenter trial of 234 patients who were studied with both single photon emission computed tomography (SPECT) and CMR perfusion imaging demonstrated superior diagnostic utility for CMR perfusion at the ideal contrast dose as compared to SPECT [12]. In lower-risk populations presenting to the ED with chest pain, negative ECG, and negative serial cardiac biomarkers, CMR was performed in two separate studies evaluating a total of 192 patients who received adenosine stress and rest CMR, LV function assessment, and LGE [13, 14]. None of the patients with a normal CMR in either of these studies had any clinical events at 9-month followup. This was corroborated in a similar study of 135 low-risk patients presenting to the ED with chest pain who received adenosine stress CMR [15]. At 1 year followup, no patients with a normal stress CMR were found to have a significant coronary stenosis or cardiac event, yielding a sensitivity and specificity of 100% and 93%, respectively. Figure 1 shows an example of an adenosine stress CMR study in a diabetic patient presenting with new onset chest pain. Stress imaging demonstrates a large area of ischemia. This patient was found to have multivessel CAD.

The costeffectiveness of using stress CMR to evaluate intermediate- and high-risk patients in the ED setting was evaluated in a study of 110 patients with chest pain, negative ECG, and negative initial biomarkers, being admitted to the hospital to rule out MI [16]. These patients were randomized to obtain adenosine stress CMR while in the ED or usual inpatient care. All health care costs relating to their hospitalization and cardiac care over 30 days were included. There was no difference in 30-day outcomes between the groups. A diagnostic protocol which included stress CMR was found to reduce inpatient admissions and produced a cost savings of over 20% in this study [16]. Further cost-effectiveness studies are still needed to address the financial implications of utilizing stress CMR in lower-risk populations.

## 5. CMR following Myocardial Infarction

**Figure 2:**
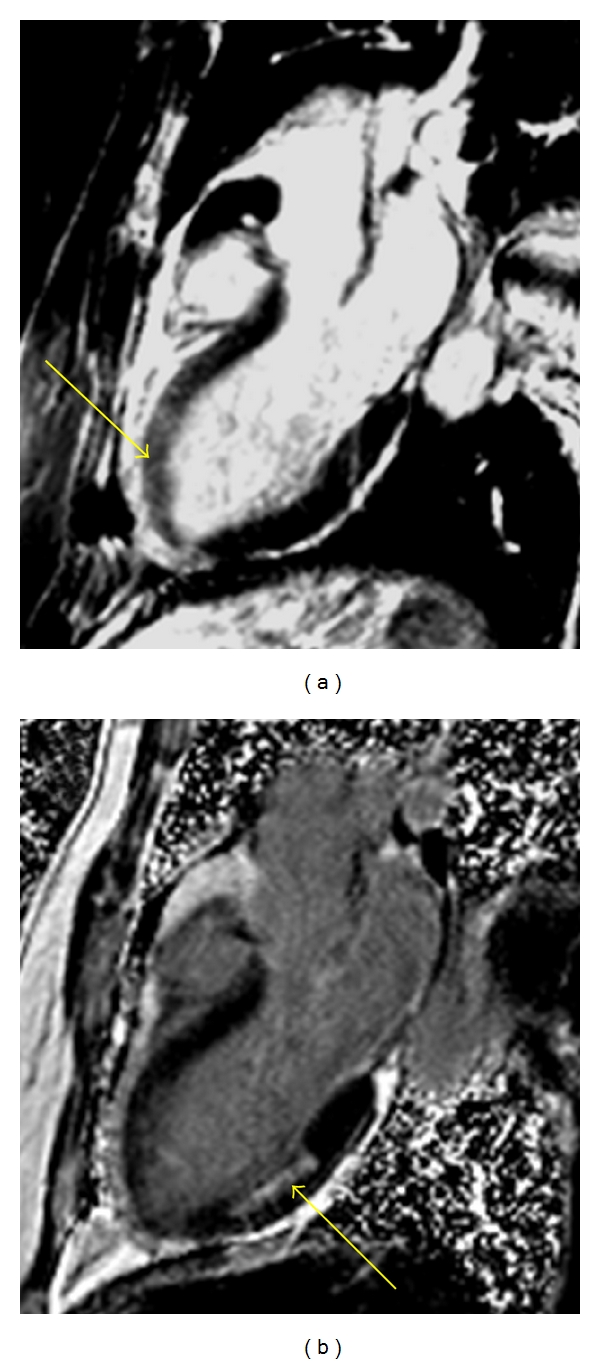
Determing acute from chronic infarcts. This patient had a CMR shortly after presenting with acute-onset chest pain. *T*_2_-weighted imaging (a) reveals a bright signal in the mid-anteroseptum and apex, signifying edema, a marker of acute myocardial injury. Late gadolinium enhanced (LGE) imaging (b) shows an infarct in the mid-inferolateral wall, but minimal enhancement in the anteroseptum or apex. This patient had a severely stenosed mid-LAD as well as an occluded obtuse marginal branch of the circumflex coronary artery. CMR in this case was able to determine the culprit vessel.

For those patients who present early after symptom onset with ST elevation MI (STEMI) or without ST elevation, but with elevated cardiac biomarkers (non ST elevation MI, or NSTEMI), an early invasive strategy with cardiac catheterization is generally recommended [17]. In these situations, it is usually not helpful to perform noninvasive cardiac testing prior to catheterization, because such tests could delay treatment and would not be expected to affect short-term patient management. CMR can be performed safely in MI patients, even immediately after percutaneous intervention [18, 19], add important prognostic information, diagnose post-MI complications, and aid in deciphering the etiology of cardiac injury in patients who present with STEMI, but who are found to have normal coronary arteries at catheterization. Figure 2 shows images from a patient who had a CMR study shortly after presenting with acute chest pain. In this case, edema imaging enabled appropriate determination of the LAD as the culprit vessel, even though there was no LGE in this region. The LGE in the lateral wall was from a prior myocardial infarction. 

### 5.1. Prognosis

Left ventricular function has long been known to predict outcomes after MI. Impaired LV systolic function and an elevated LV end-systolic volume (ESV) are both powerful independent predictors of increased mortality [20–24]. CMR has become the gold standard for accurate quantification of left ventricular function and volumes. In contemporary CMR studies of post-MI patients, LVEF and LVESV are consistently predictive of outcomes; however, multiple studies have shown that other CMR-based parameters of infarction, such as infarct size, microvascular obstruction (MO), and myocardial salvage are more powerful predictors of outcomes than simple LV function or volume assessments [25–27].

### 5.2. Infarct Size

CMR-based assessment of infarct size using late gadolinium enhancement (LGE) is well validated and has been shown to be highly predictive of cardiac events at followup. Yokota et al. evaluated 86 patients with prior MI by CMR and found that infarct size by LGE was a better predictor of cardiac events than LVEF, LVESV or LVEDV, at 20 month follow up [27]. Cheong et al. followed a cohort of 857 patients with and without coronary artery disease for a median of 4.4 years and demonstrated that LGE, LVEF and heart failure symptoms were independent predictors of mortality [28]. Interestingly, patients with LGE and an ejection fraction more than 50% had the same outcomes as those with an LVEF less than 50% without LGE [28]. A study by Larose et al. investigated 103 acute ST elevation MI (STEMI) patients by CMR within 12 hours of PCI, after which they received a 6-month CMR and were followed for clinical events for an average of 2.4 years [29]. It was demonstrated that LGE measured immediately after revascularization strongly and independently predicted 6-month LVEF and long-term major cardiac events better than LVEF or clinical parameters. This was corroborated in a study of 122 STEMI patients who received CMR after revascularization and were followed for 2 years. Infarct size was again a stronger indicator of worsened LV function and clinical outcomes at followup than were baseline measurements of LV systolic performance [30]. 

### 5.3. Microvascular Obstruction

Microvascular obstruction (MO) refers to severe capillary and endothelial damage induced by prolonged ischemia, which prevents blood flow into the infarcted core after reperfusion. The extent of MO usually peaks between 2 hours to 2 days after reperfusion occurs [31–33], after which it tends to be stable in size until at least day 9 [34]. Thereafter areas of MO involute and regress and are rarely seen on followup CMR beyond 1 to 2 months after MI.

CMR has become the gold standard for assessment of MO, due to its excellent spatial resolution and tissue characterization ability. MO can be assessed by CMR using two separate techniques. Early MO is visualized early after contrast injection using first-pass perfusion. Typical protocols using this method define MO as hypoenhancement of the myocardium persisting longer than 1 to 2 minutes after initial contrast injection. The second technique collects late LGE images 10 to 15 minutes after contrast injection, and MO appears as an area or areas of hypoenhancement encompassed within a core of enhanced infarcted myocardium, often extending from the subendocardium. This latter method in the literature is sometimes termed persistent or late MO. Both methods of MO assessment have been validated, and there is not yet a clear consensus as to which method should be the preferred technique. Early MO has been shown to be predictive of long-term infarct size, infarct transmurality, LV function, and LV remodeling and is also a powerful independent long-term prognostic indicator of hard cardiac events even after controlling for infarct size [26, 35]; however, timing of the CMR examination after MI is important when assessing MO using first-pass perfusion. Early MO was more often seen when CMR was performed 2 days after MI than on days 7 or 9 but was not predictive of long-term functional or clinical outcomes until the later time points [34, 35]. Late MO has also been shown to lend additional prognostic information beyond that of infarct size or transmurality. In a study of 110 MI patients, Hombach et al. found that late MO independently portended an increase in cardiac events at 8-month followup and gave additional prognostic information compared to infarct size alone [36]. This was corroborated in a CMR study of 67 STEMI patients followed for 14 months, in which late MO was a better predictor of adverse cardiac events than infarct size or baseline ejection fraction [37]. Data comparing early and late MO are sparse, but, in a study by Nijveldt et al., 63 acute MI patients who received PCI and optimal medical management were followed by CMR with assessment of early, mid-, and late MO 4 to 7 days after MI and followup CMR at 4 months. In this study, late MO was a better predictor of follow up LV ejection fraction, LV end-diastolic volume, and LV end-systolic volume than early MO [38].

### 5.4. Area at Risk/Myocardial Salvage

The region of ischemic myocardium within the perfusion bed of an occluded coronary artery is the “area at risk” of infarction and is potentially salvageable with appropriate and timely intervention. The area at risk, as measured by *T*_2_-weighted CMR, correlates very well to the area at risk determined histologically with fluorescein staining [39]. Similarly, *T*_2_-weighted *in vivo* CMR measurement of the area at risk 2 days following MI showed excellent correlation to the area at risk determined by fluorescent microspheres at the time of coronary occlusion [40]. 

Accurate assessment of both infarct size and the area at risk allows noninvasive measurement of a potentially clinically important parameter: the amount of myocardial salvage. The myocardial salvage index (MSI) is a measure of the myocardium that has been spared infarction, presumably due to a given procedure or treatment, and has important prognostic significance. CMR trials of STEMI patients undergoing PCI have repeatedly shown that an increase in the time from symptom onset to PCI leads to a significant incremental increase in infarct size and microvascular obstruction, with a corresponding decrease in myocardial salvage, while the extent of myocardial edema, or area at risk, remains unchanged [25, 41]. A trial of 208 STEMI patients who underwent PCI within 12 hours of symptom onset demonstrated that patients with an MSI above the study median experienced significantly fewer death, reinfarction, or heart failure events at 6 months when compared with those with an MSI below the median (2.9% versus 22.1%, *P* < 0.001) [25]. MSI was found to be a better predictor of outcome on multivariate regression than infarct size, MO, LVEF, TIMI flow-grade after PCI or ST segment resolution. Because of the unique ability of *T*_2_-weighted CMR to detect myocardial salvage and predict adverse events, it may become an extremely valuable surrogate measure of outcome in studies of reperfusion therapy. 

### 5.5. Right Ventricular Infarction

Another prognostic indicator after MI is the presence of right ventricular (RV) infarction. RV infarction has been associated with an increased risk of cardiogenic shock, atrioventricular conduction block, and rupture of the interventricular septum [42]. In-hospital mortality after RV infarction has been reported to be as high as 50% but, among survivors to hospital discharge, does not seem to predict long-term outcomes [43]. CMR has been demonstrated to be much more sensitive for detecting small or medium RV infarcts than physical exam, ECG with right-sided leads, or echocardiography [44, 45]. RV infarction, as seen by LGE of the RV after MI, was shown to be predictive of RV dilation at 6-month followup [46]. Furthermore, a recent CMR study of 50 MI patients undergoing successful PCI and followed for an average of 32 months reported RV involvement by LGE in 47% and 65% of inferior and anterior MIs; respectively, and found that RV infarction was a significant independent predictor of cardiac events (odds ratio: 15.8; 95% CI: 4–63), even after controlling for LV infarct size and location [45].

### 5.6. Postinfarct Complications

**Figure 3:**
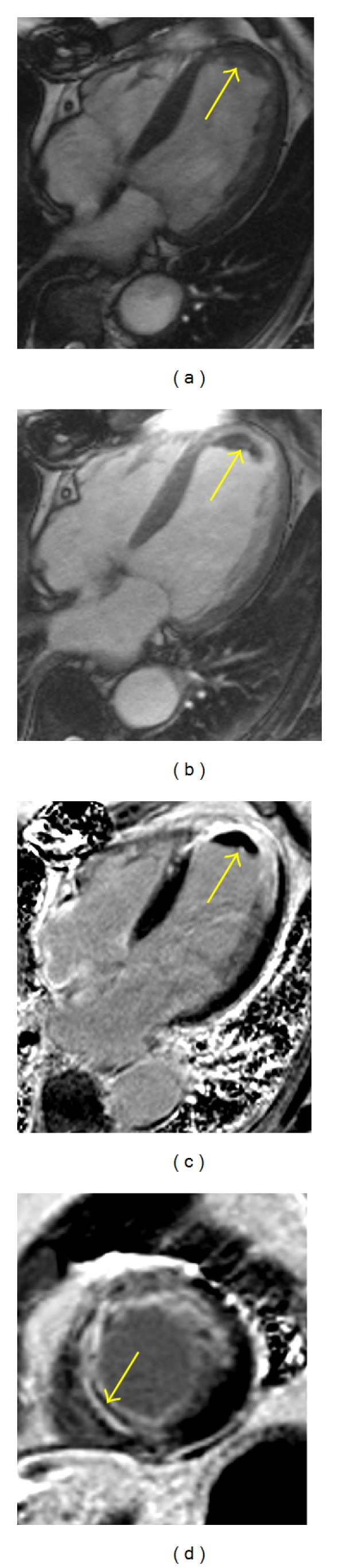
CMR imaging after acute MI. The tissue characterization abilities of CMR are demonstrated in these frames, all of which were taken from a patient who presented late after an ST elevation MI. A 4-chamber cine steady-state free precession (SSFP) image is shown in (a). Prior to contrast, it is difficult to discern whether there is thrombus in the apex. A postcontrast 4-chamber SSFP image (b) provides greater contrast between the slightly enhancing myocardium and nonenhancing thrombus at the apex. This difference is even more clear with late gadolinium-enhanced (LGE) imaging (c)-(d), which clearly demonstrates the black thrombus at the apex adjacent to the transmural apical infarct seen in white. An apical short axis view (d) reveals an extensive infarct with an area of microvascular obstruction visualized in the septum (arrow).

Due to CMR's unique tissue characterization abilities and spatial resolution, it is an ideal imaging modality for assessing complications arising after MI. CMR is widely considered the gold standard for analyzing both regional and global ventricular function after MI [47] and can comprehensively evaluate both systolic and diastolic function [48]. CMR can also readily detect ventricular wall pathology such as ventricular septal defects or free-wall aneurysms and can differentiate aneurysms from pseudoaneurysms, with its accompanying treatment implications. Visualization of LGE within the papillary muscles is indicative of infarction and has been reported in post-MI CMR studies to occur in 26 to 53% of patients [36, 49]. In a study of 60 patients with old MI, bilateral papillary muscle infarction was seen by LGE in 17% of cases and was associated with a higher incidence of severe mitral regurgitation and LV remodeling than in those with unilateral or no papillary muscle infarction [49]. Inflammatory pericarditis is visualized by LGE of the pericardium with high sensitivity and specificity after MI [50] and has been reported to occur in as many as 40% of STEMI patients [36]. CMR can also be beneficial in the evaluation of intraventricular thrombi. The presence of intraventricular thrombi has been reported in 7 to 29% of patients with decreased ejection fraction, depending on the population studied [51, 52], and is associated with a higher risk of thromboembolic events, including stroke [51, 53]. The most prominent risk factors for the development of LV thrombus include anterior infarction, infarct size, LV aneurysm, and decreased ejection fraction [52, 54]. A CMR protocol which includes LGE assessment has been shown to more accurately diagnose thrombi within the ventricle than transthoracic echocardiography (TTE) or transesophageal echocardiography (TEE). In a multimodality imaging, study of 361 patients with ischemic cardiomyopathy undergoing cardiac surgery, the presence of LV thrombus by CMR, TTE, and TEE were compared to pathology and were found to have sensitivities and specificities of 88% and 99% for CMR, 23% and 96% for TTE, and 40% and 96% for TEE, respectively [51]. While contrast echocardiography nearly doubles the sensitivity of noncontrast echo, mural, and small apical LV thrombi are still frequently missed [55]. Recently, the use of prolonged inversion times (600 ms) during LGE imaging has been shown to increase the sensitivity of LGE for detecting thrombus even further [52]. Figure 3 shows images from a patient who presented late after an STEMI demonstrating a large area of LGE and the presence of an apical thrombus.

## 6. Myocardial Injury with Normal Coronary Arteries

**Figure 4:**
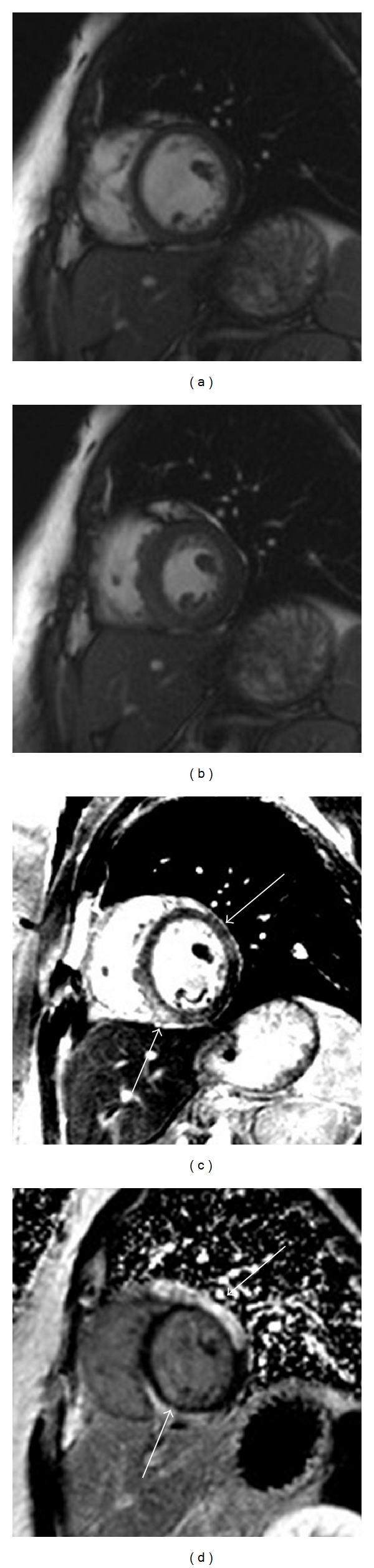
Myocardial infarction with normal coronary arteries. Images from a patient presenting with chest pain, elevated troponin but normal coronary arteries. Cine-SSFP images at (a) diastole and (b) systole demonstrate a wall motion abnormality in the inferoseptum and anterolateral walls. *T*_2_ weighted imaging (c) shows focal areas of edema in these regions. (d) LGE imaging demonstrates epicardial-delayed enhancement. These findings are consistent with the diagnosis of myocarditis.

An estimated 9–14% of patients who are diagnosed with STEMI will be found to have normal coronary arteries at cardiac catheterization [56]. These patients are at similar risk for mortality as those with CAD and often have an underlying pathology which can be difficult to diagnose. CMR is uniquely able to shed light on the underlying pathology in the majority of patients with MI with normal coronary arteries. The pattern of late gadolinium enhancement and myocardial edema in these patients is often helpful in differentiating the cause of injury. Ischemic necrosis begins in the subendocardium before extending to the epicardium; thus, sparing of the subendocardium in infarction is extremely rare. Conversely, subendocardial sparing is common in many nonischemic cardiomyopathies. Although myocarditis can present with varied LGE patterns, the classical appearance consists of a midwall or epicardial stripe of enhancement, the location of which can be indicative of the viral etiology [57, 58]. Figure 4 shows CMR images from a patient presenting with acute chest pain and a troponin elevation. Edema imaging indicated acute inflammation in the inferoseptum and anterolateral walls. LGE imaging shows epicardial scarring associated with myocarditis. Tako-Tsubo's (stress-induced) cardiomyopathy, on the other hand, does not lead to scar formation or LGE. In a study of 1345 patients with diagnosed STEMI, 127 (9.5%) were found on angiography to have no coronary artery disease [59]. CMR in these patients had a 75% diagnostic yield and differentiated 31% as myocarditis, 31% as Tako-Tsubo cardiomyopathy, and 29% as STEMI without an angiographic lesion. This was corroborated in study of 60 patients with troponin-positive chest pain, but unobstructed coronary arteries, which also found that CMR was able to provide a specific diagnosis in the majority of patients, diagnosing 50% of these cases as myocarditis and 11.6% as MI [60]. Myocardial edema, as seen with *T*_2_-weighted CMR, can help differentiate acute from chronic episodes of both ischemic and nonischemic events, and has been found to be present in acute episodes of Tako-Tsubo's cardiomyopathy [61, 62], myocarditis [63], cardiac sarcoid [64], pulmonary hypertension [65], acute transplant rejection [66], and recent cardiac surgery.

## 7. Comparative Effectiveness of CMR in ACS

In the current era of cost awareness, there is an appropriate focus on the ability of an imaging modality to improve cost and real patient outcomes when compared with other available diagnostic strategies. As already discussed, there is a relatively large body of evidence demonstrating that CMR offers excellent diagnostic and prognostic data in ACS patients and that it compares favourably to other imaging modalities. It has also been found to be cost effective in a selected population of patients presenting to the ED with suspected ACS [16]. CMR can play a key role in appropriately determining which patients with chest pain need to be admitted for further management and which patients can be safely discharged to home; this could reduce costs related to unnecessary inpatient care. There is still a great need, however, for further studies to clarify CMR's ability to directly change management, improve meaningful patient outcomes, and reduce overall costs of ACS care in the ED.

## 8. Conclusions

CMR has emerged as a robust diagnostic imaging modality capable of providing a comprehensive cardiac evaluation for patients with ACS. CMR is widely regarded as the reference standard for assessment of cardiac structure and function. In patients with acute myocardial infarction, CMR provides powerful diagnostic and prognostic information, allowing evaluation of cardiac function, infarct size and location, infarct complications, the extent of edema or area at risk, the amount of myocardial salvage, and the status of the microvasculature—all in a single scan. CMR has been shown to be safe, even immediately after PCI in STEMI patients [18, 19], and a comprehensive exam can typically be performed in less than 45 minutes without exposing the patient to radiation. For those with normal coronary arteries in the setting of MI, CMR provides clues to the underlying diagnosis in the majority of cases. For patients presenting to the emergency department with symptoms and risk factors concerning for ACS, but without evidence of MI, a comprehensive CMR study can also include stress perfusion imaging, allowing accurate functional assessment of the coronary circulation, which has been validated in both low and high risk study populations. *T*_2_-weighted imaging of edema and late gadolinium enhancement have the ability to detect infarction and severe ischemia early in the course of MI, even before cardiac biomarkers become elevated, and can differentiate acute from chronic infarction. Importantly, adding CMR to the emergency physician's tool belt has been shown to improve diagnostic accuracy compared to current standards of care and to be cost effective in certain populations. Taken together, CMR provides a wealth of meaningful diagnostic and prognostic data for a wide range of patient populations with suspected or confirmed acute coronary syndrome.

## Abbreviations

ACS: Acute coronary syndrome
CAD: Coronary artery disease
CMR: Cardiac magnetic resonance imaging
ECG: Electrocardiogram
ED: Emergency Department
HF: Heart failure
LGE: Late gadolinium enhancement
LV: Left ventricle
LVEF: Left ventricular ejection fraction
MI: Myocardial infarction
NSTEMI: Non-ST elevation myocardial infarction
PCI: Percutaneous coronary intervention
RV: Right ventricle
STEMI: ST elevation myocardial infarction
UA: Unstable angina.
